# A novel pathogenic variant in the *LRTOMT* gene causes autosomal recessive non-syndromic hearing loss in an Iranian family

**DOI:** 10.1186/s12881-020-01061-7

**Published:** 2020-06-09

**Authors:** Akram Sarmadi, Samane Nasrniya, Maryam Soleimani Farsani, Sina Narrei, Zahra Nouri, Mahsa Sepehrnejad, Mohammad Hussein Nilforoush, Hamidreza Abtahi, Mohammad Amin Tabatabaiefar

**Affiliations:** 1 Department of Genetics and Molecular Biology, School of Medicine, Isfahan University of Medical Sciences, Isfahan, Iran; 2 Genetics Department, Erythron Pathobiology and Genetics lab, Isfahan, Iran; 3 Department of Biotechnology, Faculty of Advanced Sciences and Technologies, University of Isfahan, Isfahan, Iran; 4 Department of Medical Biotechnology, School of Advanced Technologies in Medicine, Tehran University of Medical Sciences, Tehran, Iran; 5 Department of Otolaryngology, Al-Zahra Hospital, Isfahan University of Medical Sciences, Isfahan, Iran; 6 Pediatric Inherited Diseases Research Center, Research Institute for Primordial Prevention of Noncommunicable Disease, Isfahan University of Medical Sciences, Isfahan, Iran; 7 GenTArget Corp (GTAC), Deputy of Research and Technology, Isfahan University of Medical Sciences, Isfahan, Iran

**Keywords:** Frameshift mutation, Hearing loss, Iran, *LRTOMT*, Pathogenic variant, Whole exome sequencing

## Abstract

**Background:**

Hearing loss (HL) is the most common sensorineural disorder with high phenotypic and genotypic heterogeneity, which negatively affects life quality. Autosomal recessive non-syndromic hearing loss (ARNSHL) constitutes a major share of HL cases. In the present study, Whole exome sequencing (WES) was applied to investigate the underlying etiology of HL in an Iranian patient with ARNSHL.

**Methods:**

A proband from an Iranian consanguineous family was examined via WES, following *GJB2* sequencing. WES was utilized to find possible genetic etiology of the disease. Various Bioinformatics tools were used to assess the pathogenicity of the variants. Co-segregation analysis of the candidate variant was carried out. Interpretation of variants was performed according to the American College of Medical Genetics and Genomics (ACMG) guidelines.

**Results:**

WES results showed a novel frameshift (16 bp deletion) variant (p.Ala170Alafs*20) in the *LRTOMT* gene. This variant, which resides in exon 6, was found to be co-segregating in the family. It fulfils the criteria set by the ACMG guidelines of being pathogenic.

**Conclusion:**

Here, we report successful application of WES to identify the molecular pathogenesis of ARNSHL, which is a genetically heterogeneous disorder, in a patient with ARNSHL.

## Background

Hearing loss (HL) is the most common congenital sensorineural defect affecting about 1 of 500–1000 newborns worldwide. It represents a significant global health problem [1]. HL has a wide spectrum of clinical manifestations: congenital or late onset, conductional or sensorineural, syndromic or non-syndromic [2]. Approximately, 50% of HL is related to genetic causes [2], meanwhile environmental and age-related HL account for the remaining percentage [3, 4]. Non-syndromic HL (NSHL) is responsible for 70–80% of all hereditary cases of HL. It, in turn, includes the autosomal recessive (AR) pattern (75%), the autosomal dominant (AD) (20–25%) and mitochondrial and X-linked HL (about 1%) [5–7]. Syndromic HL (SHL) accounts for the remaining 20–30% of genetic disorders in children [7]. There are about 400 types of syndromic HL [8]. HL is frequent in the Middle East and Northern African countries, with high rate of consanguinity [9, 10]. Iran, which has a consanguinity rate of about 38.6%, is an appropriate region for HL studies [11].

Mutations in one single locus, DFNB1 locus at 13q11–12 containing *GJB2 (gap junction protein β-2)* and *GJB6* genes [7], account for 50% of the ARNSHL etiology in many Western populations [12]. In Iran, the prevalence of the *GJB2* mutations is variable, depending on ethnicity and geographical location [13, 14]. The average percentage of *GJB2* mutations, as the cause of ARNSHL, in Iran is about 18.7% [15], with a higher frequency in the north (33%) and a lower frequency (4%) in the southern regions. In this region, mutations in *SLC26A4* are more frequent [16]. After the exclusion of *GJB2*, recognizing the underlying gene is difficult due to the high degree of genetic heterogeneity of HL. Therefore, whole exome sequencing would be ideal to determine HL causing mutations [17].

**Fig. 1:**
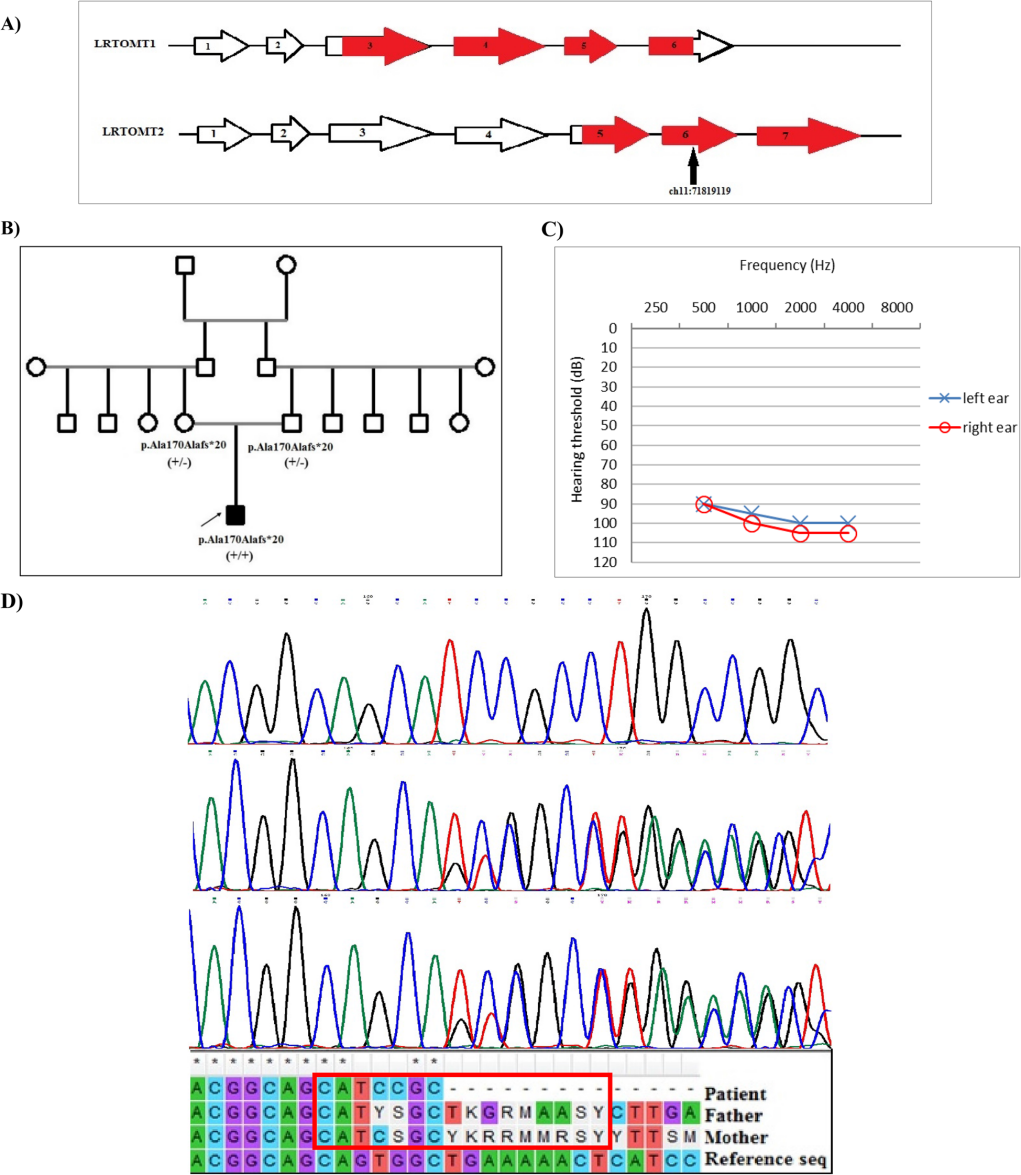
**a** Two isoforms encoded by the *LRTOMT* gene: LRTOMT1 and LRTOMT2. LRTOMT1 starts in exon 3 and LRTOMT2 starts in exon 5. CDS regions are colored red. In this transcript of *LRTOMT* (NM_001145308), LRTOMT2, starts from exon 3 and ends in exon 7. **b** Pedigree of the family. The proband is marked by an arrow. **c** Pure tone audiogram of patient. Audiogram indicate sever-to-profound hearing loss in both ears. Frequency in hertz (Hz) and the hearing threshold in decibels (dB) are shown. **d** The electropherogram of the muatation in the patient (A1), deletion of 16 bp homozygously, in his father (A2) and his mother (A3) heterozygously. In the bottom of the electropherograms, the comparison of three sequence with refrence sequence is shown. The deleted 16 bp is shown in red box

DFNB63 (OMIM 611451) was mapped to human chromosome 11q13.3-q13.4 [18–20]. This region contains the *LRTOMT* (*Leucine Rich Transmembrane and O-Methyl-Transferase*) gene [21]. *LRTOMT* is a fusion gene that has alternative reading frames and only exists in primates. Human *LRTOMT* has 10 exons, of which the first two are non-coding. Five transcripts have been reported for this gene. It has two different major protein products namely LRTOMT1 and LRTOMT2, which differ in the position of the start codons (Fig. 1a) [18]. *LRTOMT* is expressed in sensory hair cells with a fundamental role in auditory and vestibular functions [22]. LRTOMT1 is of unknown function while LRTOMT2 participates in inactivation of catecholamine neurotransmitters. Notably, there is homology between LRTOMT2 and COMT and the majority of the residues that are involved in the substrate binding region are conserved [23, 24]. Accordingly, it seems that LRTOMT2 might function as a catechol-O-methyltransferase [25]. Thus, LRTOMT2 has been named as COMT2. It catalyzes the transfer of a methyl group from S-adenosyl-L-methionine (AdoMet) to a hydroxyl group of catechols [23]. It is expressed in sensory hair cells in the inner ear. The defects in O-methyl transferase protein have been noted to cause NSHL [16]. Missense mutations cause a significant reduction in COMT2 enzymatic activity, suggesting that a defect in catecholamine catabolism underlies auditory and vestibular phenotypes [22]. The 11q13.3-q13.4 includes *FGF3* (*Fibroblast growth factor 3*) gene, too. Mutations of this gene cause a form of syndromic HL (OMIM 610706), characterized by microtia, microdontia and inner ear agenesis [26, 27]. Patients with NSHL that are due to mutations in the *LRTOMT* gene have been found to be segregating only in the Middle Eastern, which is of a high consanguineous marriage [20, 21, 26, 28–30]. The highest mutation frequency in this gene is reported in Tunisian families and then in Iranian, Turkish and Pakistani families [21, 22]. These mutations lead to severe-to-profound prelingual NSHL [18].

The recently developed next generation sequencing (NGS) technologies such as targeted NGS (TNGS), whole genome sequencing (WGS) or whole exome sequencing (WES) have revolutionary improved disease diagnosis [31] and discovery of novel disease-causing genes and variants [32]. Recent advances in DNA enrichment and NGS methods have provided the opportunity for rapid and cost-effective analysis to identify pathogenic mutations in HL patients [33]. WES is an acceptable method with efficient strategy to recognize disease-causing mutations in genetically heterogeneous diseases. In this method, single nucleotide variants (SNVs), small insertion/deletions, and sometimes structural changes such as copy number variations (CNVs) can be diagnosed [34, 35].

The aim of this study was to identify the molecular pathology of congenital HL in a four-year-old boy using WES, which led to the identification of a novel frameshift mutation.

## Methods

### Subject and clinical evaluations

A four-year-old boy with congenital HL from an Iranian consanguineous family (first cousins) with no history of HL was ascertained (pedigree is shown in Fig. 1b). Comprehensive family history, audiological testing such as play audiometry, tympanometry, acoustic stapedial reflex, transient /distortion product oto acoustic emission (TE/DPOAEs), auditory brainstem response (ABR), auditory steady state response (ASSR) and physical examination were performed. The parents signed written informed consent following pre-test genetic counseling. Clinical examinations did not reveal any symptoms of syndromic form of HL. The patient received cochlear implant at the age of 1 year and 5 months.

Auditory and speech performance in these cases were evaluated using the categories of auditory performance (CAP) [36] scale and speech intelligibility rating (SIR) [37] scale. These scales are reliable for measuring the outcome of cochlear implantation [38, 39]. CAP consists of 8 categories, ranging from “no awareness of environment” (CAP score 0) to “use of telephone with known users” (CAP score 7) and SIR consist of 5 categories, ranging from “unintelligible speech” (SIR score 1) to “speech intelligible to all listeners” (SIR score 5).

### Molecular study

#### DNA extraction, *GJB2* sequencing

Genomic DNA was extracted using Prime Prep Genomic DNA Extraction kit from blood (GeNet Bio, Korea), according to the manufacturer’s instruction. DNA purity and concentration was determined using the Nanodrop 2000 spectrophotometer (Nonodrop 2000 Thermo Scientific, USA) and its quality was checked on 1% agarose gel.

Sanger sequencing was performed in order to exclude *GJB2* mutations and the following primers were used: F: 5′-CTCCCTGTTCTGTCCTAGCT-3′ and R: 5′-CTCATCCCTCTCATGCTGTC-3′ [40]. Because there was no positive family history of HL or other cognitive disorders in the family, linkage analysis was not sought.

#### Whole exome sequencing and bioinformatic analysis

The Sample was sent to Macrogen (South Korea) (https://www.macrogen.com/) and was subjected to WES using the Novaseq 4000 platform (Illumina, San Diego, CA, USA) with 151-bp paired-end reads. In summary, genomic DNA was fragmented to prepare Illumina library and fragments were captured to target all exons, splicing sites, and flanking intronic sequences of all genes (Agilent SureSelect V6 post). All fragments were amplified and then, sequencing was performed (the mean depth of coverage was 100X for greater than 92% of the sequences). For the studied sample, 57,006,242 reads were produced, and total read bases were 8.6G bp. The GC content was 52.01% and Q30 was 93.47%. After performing WES, the released raw data were converted to the FASTQ file. Bioinformatic analysis included GATK (Genome Analysis Toolkit) (https://gatk.broadinstitute.org/) for variant calling, BWA (Burrows-Wheeler Aligner) (http://bio-bwa.sourceforge.net/) for genome alignments and variant detection (hg19, NCBI Build 38) and Picard to mark duplicate reads were used. Variant filtering was performed based on Homozygous missense, start codon change, splice site, nonsense, stop loss, and indel variants with MAF < 1% in databases such as: dbSNP version 147, 1000 genomes project phase 3 database (https://www.internationalgenome.org/), NHLBI GO exome sequencing project (ESP) (https://evs.gs.washington.edu/), exome aggregation consortium (ExAC) (http://exac.broadinstitute.org) and Iranome (http://www.iranome.ir/).

After the filtration, the reported frameshift variant was evaluated by different in silico software tools such as PROVEAN (http://provean.jcvi.org/), PANTHER (http://www.pantherdb.org/), MutationTaster (http://www.mutationtaster.org/), SIFT (https://sift.bii.a-star.edu.sg/) and CADD (https://cadd.gs.washington.edu/) to predict its deleterious effect on protein in terms of function. Furthermore, the degree of conservation of this variants was assessed using NCBI BLAST of several vertebrate species [41].

#### Variant confirmation

The candidate variant was confirmed using bidirectional Sanger sequencing. Then, co-segregation analysis was performed using exon-specific custom primers to examine segregation of genotype and HL phenotype among the family members. PCR amplification and sequencing of this variant were performed using the forward primer: 5′-GCATCCATCTCCCATGTCTT-3′ and the reverse primer: 5′-CACCATCCAGCATCAGTC-3′ in exon 6. Chromatograms were compared with reference sequence (NM_001145308), encoding a 291 residue protein (NP_001138780.1), using SeqMan software version 5.00© (DNASTAR, Madison, WI, USA). Next, this variant was investigated in the Human Gene Mutation Database (HGMD) (http://www.hgmd.cf.ac.uk/) and the literature to seek the novelty of the variant or its association with HL. Variant nomenclature was based on Human Genome Variation Society (HGVS) [41]. The American College of Medical Genetics and Genomics (ACMG) guidelines were also used to classify this variant [42]. The MEGA6 software was used to check the conservation of the mutated region in several species.

## Results

### Clinical evaluations

The proband was a four-year-old boy who showed bilateral profound NSHL, according to the audiological evaluations (Fig. 1c). Syndromic forms of HL were ruled out in this family, based on the history and clinical examination in the patient. The proband was born to a consanguineous first-cousin couple after a full-term natural delivery He showed no developmental delay or developmental regression, based on medical reports and examinations during pre-test genetic counseling. No genetic disease other than HL was evident in the related pedigree (Fig. 1). The CT scan results of Temporal Bone were normal in the patient.

Results of auditory and speech performance indicate good outcome of cochlear implantation in the patient after 3 years with CAP score of 6 (understanding conversation without lip reading) and SIR score of 5 (speech is intelligible to all listener).

### Molecular findings

Direct sequencing of the coding exon of the *GJB2* gene did not show any mutation. WES was applied and totally 672,262 variants were detected; one of them met the criteria for further analyses.

As a result of WES, a homozygous deletion of 16 nucleotides c.509_524del **CAGTGGCTGAAAAACT** (p.Ala170Alafs*20) in the *LRTOMT* gene was found. It causes frameshift in exon 6 of this gene. It creates alternation of 20 amino acids downstream of the deletion and leads to an early stop codon, resulting in a truncated protein with 170 residues (versus 291 residues in the intact protein). This deletion mutation was assessed as being deleterious by Mutation Taster as well as several other prediction tools such as SIFT, PROVEAN, PANTHER (Table 1). The frameshift variant was absent from dbSNP version 147, 1000 genomes project phase 3, NHLBI GO ESP, ExAC, Iranome, HGMD and Clinvar databases. It was not found in the literature, either. Ala170 and the following 20 amino acids that are modified in the mutated protein are located in a highly conserved residue of LRTOMT in multiple-species alignment (Fig. 3). It has been conserved among several species including *Pan troglodytes*, *Macaca mulatta, Mus musculus, Rattus norvegicus* and *Xenopus tropicalis*.

**Table 1:** In silico analysis of identified variants in the *LRTOMT* gene

Variant/genomic Location	Exon	Amio- acid alteration	Database	Software	SIFT	Mutation Taster2.0	PROVEAN	PANTHER
**c.509_524del** **(CAGTGGCTGAAAAACT)** **Frameshift mutation** **(Long InDel)**	6	A170Afs*20	1000 G	state	Deleterious	Disease-Causing	Deleterious	Deleterious
			Not found					
			ExAC	Score	0.894	NA	−4.709	–
			Not found					

The variant co-segregated with the disease in the family: heterozygous in parents, but homozygous in the patient who was the only child of the family (Fig. 1d).

According to the ACMG guideline, that its evidence is describe below, this variant is classified as a **pathogenic** variant (1 very strong, 2 Moderate and 1 Supporting criteria):

- It is a frameshift variant (a null variant) (**PVS1**).
- It is located in a mutational hot spot and/or critical and well-established functional domain (**PM1**).
- This variant is absent from controls (or at extremely low frequency if recessive) in Exome Sequencing Project, 1000 Genomes Project, Exome Aggregation Consortium and Iranome (local database) (**PM2**).
- Multiple lines of computational evidence supported the deleterious effect of the variant on the gene or gene product (conservation, evolutionary, splicing impact, etc.) (**PP3**).

### The 3-D structure of the LRTOMT protein (wild-type and mutated forms)

**Fig. 2:**
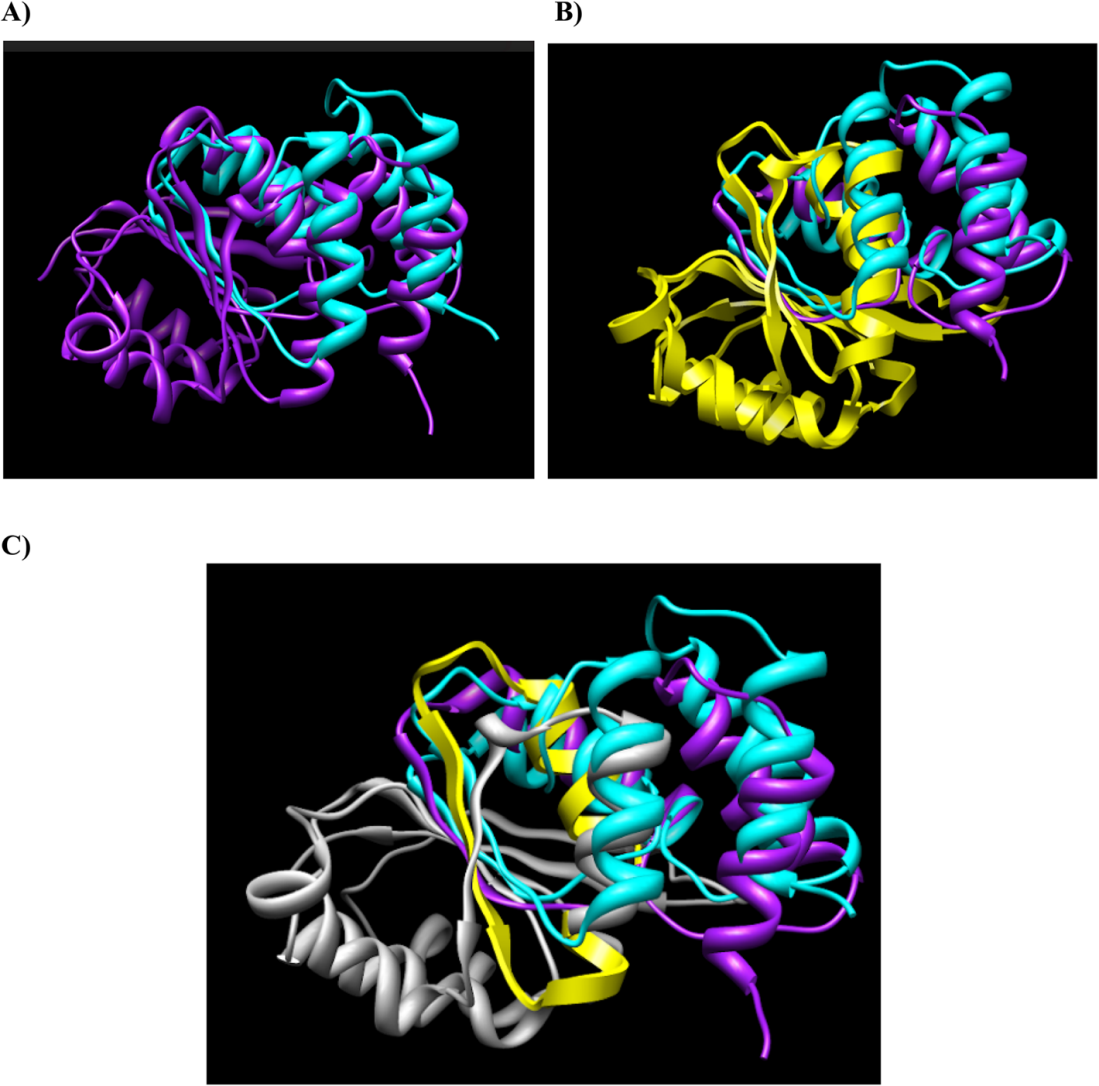
Protein structure modeling of wild-type and mutated LRTOMT. **a** the merged image of wild-type LRTOMT is shown in purple and mutanted LRTOMT in cyan. A part of the amino acid sequence has been eliminated in the mutated protein compared to the wild type protein (**b**) the catechol-O-methyltransferase domain, as a functional domain, is shown in yellow and in the figure (**c**) the modified COMT domain in the mutanted protein is defined in gray. The affected amino acids (residue 170–291) are a part of catalitic domain

PDB files of the wild type and mutated protein were generated from protein structure prediction server (http://ps2.life.nctu.edu.tw/). Then, UCSF Chimera version 1.5.3 (https://www.cgl.ucsf.edu/chimera/) was used to construct the 3D structure of both wild-type and the mutant forms of LRTOMT. The pictures show a comparison between wild-type and mutant protein, the catalytic domain (catechol-O-methyltransferase) that is modified in the mutant protein (Fig. 2).

## Discussion

Hearing loss (HL) is a common sensorineural disorder, with an incidence of 1 in every 500–1000 children [43]. NSHL is a highly genetically heterogeneous neurosensory disorder with over 163 known genes (http://hereditaryhearingloss.org/) [44]. Although mutations in the *GJB2* gene has been known to be the most common cause of ARNSHL in Iran (about 18.5% of HL cases), the role of other genes is remains to be illuminated [15, 45, 46]. Lack of accurate DNA diagnostics represents a real challenge for NSHL diagnosis and genetic counselling [47]. In this regard, NGS has addressed this problem with generation of huge data from our genes in a rather short time [48, 49].

In Iran, HL ranks second after intellectual disability [15]. To date, few studies have been performed on *LRTOMT* mutations in Iran and frequency of these mutations is not exactly known among Iranian ARNSHL patients [28, 50]. So far, about 20 pathogenic mutations have been reported in the *LRTOMT* gene (Table 2). The human *LRTOMT* gene (the DFNB63 locus) is located on the chromosome 11q13.2-q13.4 and encodes the LRTOMT protein. The locus DFNB63 was firstly mapped to the long arm of chromosome 11 by *Kalay* et al*,* who reported a five-generation Turkish family to be linked to this region [18]. Findings indicated that mouse *Lrrc51* and *Tomt* are two separate genes encoding 2 different proteins, and that human *LRTOMT* gene is a larger fusion gene with two different transcripts [21]. The LRTOMT2 (residues 79 to 291) protein has a transmembrane catechol-O-methyltransferase (COMT) domain and is also known as COMT2, which is highly expressed in sensory hair cells and the vestibular organs of the inner ear [22]. The most important COMT domain is identified in the generic COMT protein that catalyzes the transfer of a methyl group getting from S-adenosyl methionine to catecholamines. It is associated with inactivation of catecholamine neurotransmitters like norepinephrine, dopamine and epinephrine [23, 57]. In a study in 2008, the mouse model of *Comt2* mutation was generated and it was shown that mutation in this gene leads to profound HL and vestibular defect [22]. By using a zebrafish model, *Erickson* et al in 2017 showed that the defect in auditory and vestibular systems due to mutations in *LRTOMT* gene. It led to a lack of mechanotransduction (MET), a process in which sensory hair cells convert mechanical energy such as vestibular and auditory stimulation to electrical signals [58]. The *TMC* (*Transmembrane channel-like*) gene appears to be the most promising candidates to be the precursor of MET channel [59–62]. In humans, mutations in *TMC1* gene are responsible for both recessive (DFNB7/11) and dominant (DFNA36) forms of NSHL [63]. Using the mercury mutant zebrafish, as a model of DFNB63, *Erickson* reported that *LRTOMT* is required for trafficking TMC proteins to the hair bundle [58, 64].

**Table 2:** Overview of all LRTOMT mutations so far identified

Variant	Codon number	Exon number	Phenotype	Reference	Population
p.Leu16Pro	16	4	Prelingual HL	Du (2008) [22]	Iranian
p.Ala29Serfs*54	29	4	NA	Ahmed (2008) [21]	Turkish
p.Met34Ilu	34	5	HL, non-syndromic	Babanejad (2012) [28]	Iranian
p.Ser35Serfs*13	35	5	Sensorineural HL	Vanwesemael (2011) [51]	Iranian
p.Glu40Asp	40	5	Prelingual profound HL	Babanejad (2012) [28]	Iranian
p.Arg41Trp	41	5	NA	Babanejad (2012) [28]	Iranian
p.Arg52Trp	52	5	Non-syndromic HL	Wang (2017) [52]	Pakistani
p.Arg54Gln	54	5	Prelingual moderate	Ichinose (2015) [53]	Japanese
p.Arg70X	70	5	Non-syndromic HL	Riahi (2014) [54]	Iranian
p.Tyr71X	71	5	Prelingual HL	Du (2008) [22]	Iranian
p.Glu80Asp	80	5	Non-syndromic HL	Babanejad (2012) [28]	Iranian
p.Arg81Gln	81	5	Non-syndromic HL	Ahmed (2008) [21]	Tunisian
p.Arg81Trp	81	5	Non-syndromic HL	Babanejad (2012) [28]	Iranian
p.Phe83Lue	83	5	NA	Marková (2016) [55]	Czech
p.Trp105Arg	105	5	Non-syndromic HL	Ahmed (2008) [21]	Tunisian
p.Glu110Lys	110	5	Non-syndromic HL	Ahmed (2008) [21]	Tunisian
p.Tyr111X	111	5	Non-syndromic HL	Du (2008) [22]	Iranian
p.Ala170Alafs*20	**170**	**6**	**Non-syndromic HL**	**This study**	**Iranian**
p.Ilu188Thrfs*7	188	7	Prelingual moderate	Ichinose (2015) [53]	Japanese
p.Arg219X	219	7	Severe-profound NSHL	Sloan-Heggen (2016) [56]	Not defined

*NA* Not Available

In several studies, it has been reported that NSHL due to mutations in the *LRTOMT* gene are more likely to be assigned to the LRTOMT2 (COMT2) region rather than LRTOMT1 [28, 51]. These findings indicate mutations in *LRTOMT2* are associated with hair cell defects and lead to severe-to-profound NSHL [21, 29, 30, 53]. Patients with mutations in the *LRTOMT* gene have been reported exclusively from the Middle Eastern consanguineous families [19, 20, 28, 50]. The majority of reported mutations occurred in exon 5 and 7 of the *LRTOMT2* coding region. Therefore, this region might be a mutational hot spot in the *LRTOMT* gene [9, 53].

**Fig. 3:**
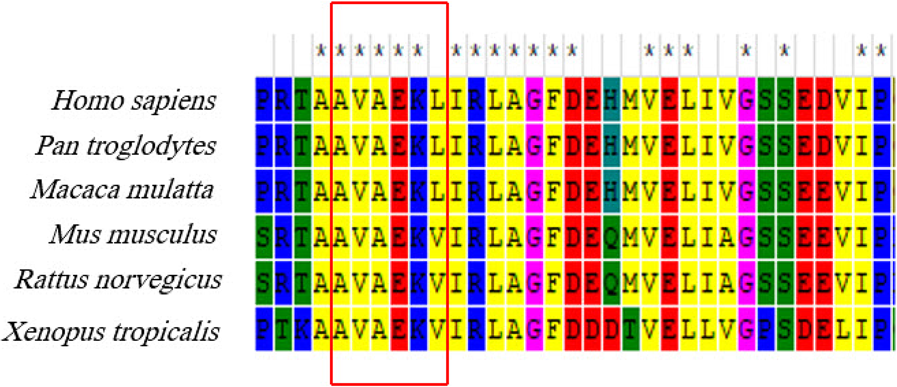
The modified region (5 deleted amino acids shown in the red box and the folowing 20 residues that are changed due to the frameshift mutation) is located in a highly conserved region among species

In this study, a consanguineous family with a son suffering from bilateral sever-to-profound HL and negative for *GJB2* gene mutations was selected for further study using WES. As a result, a novel long deletion variant (16 bp deletion) in the exon 6 of *LRTOMT* gene (NM_001145308) was found homozygously in the patient and heterozygously in the parents. It is a novel frameshift mutation (deletion of 16 nucleotides) in the *LRTOMT* gene, which has dual reading frames (ENST00000307198.7). In this transcript of *LRTOMT* gene, LRTOMT2 starts in exon 5 and ends in exon 7. The main portion of the catalytic domain (residues 79–291) of LRTOMT2 is eliminated as a result of this mutation (Fig. 2). The premature stop codon (20 codons after the position of the deletion) is predicted to result in a truncated protein with impaired function or no protein production, possibly because of nonsense mediated mRNA decay. The mutation region is a partly conserved in human and mice (Fig. 3), suggesting that the eliminated region is important for the catalytic functions of the enzyme.

Evaluation of auditory and speech performance in this study is in line with those of previous studies, suggesting that patients with *LRTOMT* mutations show good auditory performance after cochlear implant surgery [65].

## Conclusions

In conclusion, our results suggest that mutations in the *LRTOMT* gene result in alterations in the LRTOMT2 (COMT2) protein and might be involved in sever-to-profound NSHL. The gene should be studied in a larger population of families in Iran for a more thorough understanding of its role in causing HL. This study showed that exome sequencing is an efficient molecular diagnostic method for ARNSHL as an extremely heterogeneous genetic disorder.

## Data Availability

The raw datasets generated and/or analyzed during the current study are not publicly available because it is possible that individual privacy could be compromised, but are available from the corresponding author on reasonable request.

## Abbreviations

HL: Hearing loss
ARNSHL: Autosomal recessive non-syndromic hearing loss
WES: Whole exome sequencing
ACMG: American College of Medical Genetics and Genomics
AR: Autosomal recessive
AD: Autosomal dominant
LRTOMT: Leucine Rich Transmembrane and O-Methyl-Transferase
COMT: Catechol-O-methyltransferase
OMIM: Online Mendelian Inheritance in Man
WGS: Whole genome sequencing
NGS: Next generation sequencing
TNGS: Targeted next generation sequencing
SNV: Single nucleotide variants
CNV: Copy number variants
CAP: Categories of Auditory Performance
SIR: Speech Intelligibility Rating
BWA: Burrows-Wheeler Aligner
GATK: Genomic Analysis Toolkit
MAF: Minor Allele Frequency
NHLBI: National Heart, Lung, and Blood Institute
ESP: Exome Sequencing Project
ExAC: Exome Aggregation Consortium
SIFT: Scale-invariant feature transform
CADD: Combined Annotation Dependent Depletion
NCBI: National Center for Biotechnology Information
BLAST: Basic Local Alignment Search Tool
PCR: Polymerase chain reaction
HGMD: Human Gene Mutation Database
HGVS: Human Genome Variation Society
PDB: Protein data bank
MET: Mechanotransduction
